# Primary papillary carcinoma of the thymus with invasion into subcutaneous tissue through the sternum

**DOI:** 10.1186/1749-8090-9-77

**Published:** 2014-05-02

**Authors:** Yuta Ibuki, Jiro Okami, Yasuhiko Tomita, Ayako Fujiwara, Takashi Kanou, Toshiteru Tokunaga, Masahiko Higashiyama

**Affiliations:** 1 Department of General Thoracic Surgery, Osaka Medical Center for Cancer and Cardiovascular Diseases, 1-3-3 Nakamichi Higashinari, Osaka 5378511, Japan; 2 Department of Pathology, Osaka Medical Center for Cancer and Cardiovascular Diseases, Osaka, Japan

**Keywords:** Thymus, Cancer, Sternum, Surgery, Chest wall

## Abstract

Thymic carcinoma is a rare malignant neoplasm. We present a Japanese case of papillary carcinoma of thymus in a 64-year-old man that invaded into subcutaneous tissue penetrating the sternum. We describe the clinical and pathologic features of this extremely rare thymic epithelial tumor, with disease-free survival at three years of follow-up.

## Background

Thymic carcinoma is a rare tumor. The most common histological subtype of thymic carcinoma is squamous cell carcinoma, and adenocarcinoma is extremely rare. We report the case of a 64-year-old Japanese man with thymic papillary carcinoma. In this case, the tumor invaded into subcutaneous tissue by penetrating the sternum with osteolytic change.

## Case presentation

**Figure 1:**
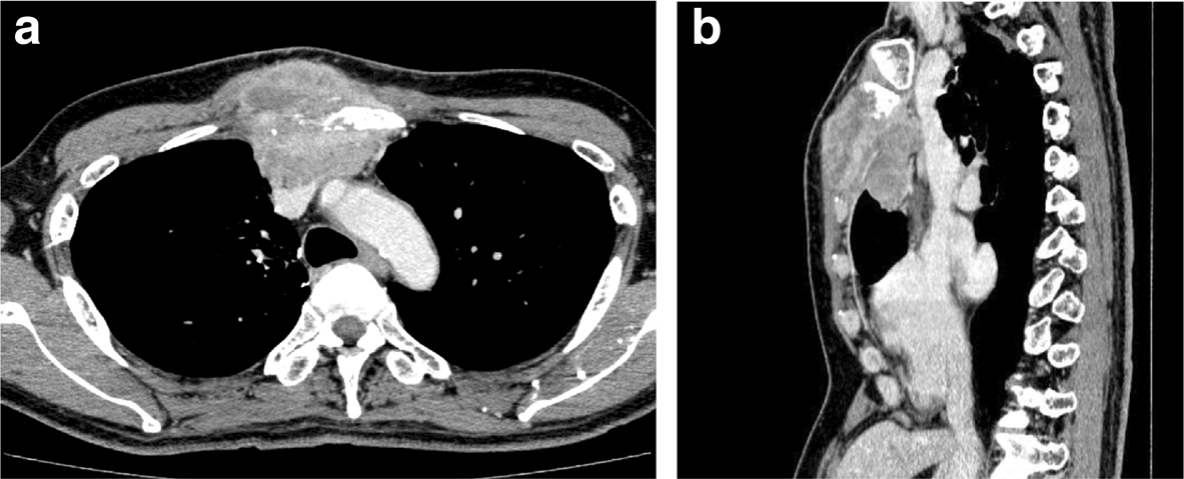
**The computed tomographic scan showed an anterior mediastinal tumor penetrating into the sternum. (a)** Horizontal section; **(b)** sagittal section.

A 64-year-old Japanese man presented with bulging of the anterior chest wall for 3 months duration. On examination, a round mass was palpable with tenderness and redness, and fixed in the upper mid- sternum. The patient had undergone wedge resection of the right upper lobe due to a spontaneous pneumothorax when he was young. Computed tomographic (CT) scan showed a 7.5 × 5.5 × 7.2 cm mass in anterior mediastinum invading into subcutaneous fat tissue through the sternum with bone destruction (Figure 1a and b). On fluorine-18-fluorodeoxyglucose (FDG)-positron emission tomography (PET), abnormal FDG uptake was observed only in the mediastinal mass with a maximum standardized uptake ratio of 15.0. Serum carcinoembryonic antigen (CEA) was remarkably elevated to be 2047.2 ng/ml but other tumor markers (AFP, beta-HCG, SCC), anti-acetylcholine receptor antibody, and an alkaline phosphatase were negative. The tumor was diagnosed to be carcinoma by fine-needle aspiration, but the definitive histology could not be determined.

The patients underwent enbloc resection of the tumor through a midline incision over the sternum and a right collar incision. The tissue around the tumor was mobilized in a circumferential manner, and an upper half of the sternum including terminal 3–5 cm of the clavicle and the upper three ribs on the right side, medial end of the left clavicle, and adjacent subcutaneous tissue and the skin. The great vessels and the pericardium were free from the tumor. Because upper lobes of both lungs were adherent to the mediastinal pleura at the tumor site, wedge resections were performed. Regional lymph node exploration revealed no nodal involvement of the tumor cells. The sternal defect was not reconstructed. The postoperative course was uneventful and the patient was discharged on the 14^th^ day after surgery. Paradoxical respiratory movement of the chest was not observed. Serum CEA level returned to normal 3 months after surgery.

**Figure 2:**
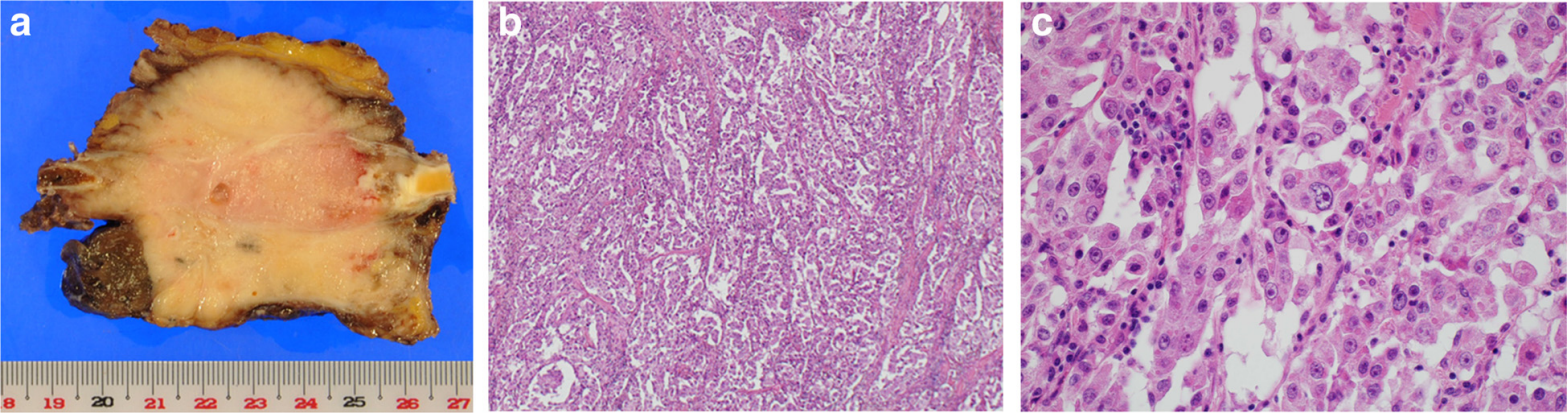
A gross specimen of the thymic tumor (a) and pathological findings (Hematoxylin and eosin stain, original magnification x40 for b and x200 for c).

Macroscopic examination of the tumor penetrating the sternum was not encapsulated but showed a vaguely infiltrative border with extension into mediastinal tissue and subcutaneous fat (Figure 2a). Histological examination revealed that the tumor cells proliferate in papillary clusters separated by fibrovascular septa (Figure 2b and c). Tumor cells are round to oval with eosinophilic cytoplasm. The nuclei of tumor cells are bizarrely shaped with prominent nucleoli. Parietal involvement was positive, but histological invasion of the tumor cell into pulmonary parenchyma was not observed. The result of immunohistochemical investigations were that CEA was positive but thyroglobulin, BER-EP4, CD56, chromogranin A, and synaptophysin were negative. Based on these findings, the primary thymic papillary adenocarcinoma was diagnosed. The disease was described at stage III (T3N0M0) according to Weissferdt-Moran TNM Staging System for Thymic Carcinoma [1] and also at the same stage by Masaoka staging system for thymoma. Any adjuvant therapy was performed for this patient because there is no evidence-based treatment for thymic carcinoma after the tumor is completely resected. The patient has been free of disease for 36 months after surgery without any other treatment.

## Discussion

Thymic carcinoma is relatively uncommon among thymic epithelial tumors, constituting approximately 10% of these lesions [2]. While most of them are squamous cell carcinoma, papillary carcinoma, one of the subtypes of thymic adenocarcinoma, is an exceedingly rare tumor and was first described in 1998 [3]. According to the histological study of 65 patients with thymic carcinoma, papillary carcinoma represents only 2 cases (3%) [4]. Recently, two of the cases with papillary thymic adenocarcinoma have been reported. One developed in a thymic cyst [5] and the other one was incidentally found in the resected specimen of type AB thymoma [6]. Contrary to the thymus, the papillary morphology is common in other organs. For differential diagnosis, the possibility of a metastatic papillary carcinoma or a papillary carcinoma in an ectopic thyroid gland should be excluded. In this case, following negative immunohistochemical staining for thyroglobulin and no subsequent malignant disease after surgery, we have confirmed the diagnosis. The clinical features and the treatment outcomes of thymic papillary carcinoma have not been well described yet. The limited available reports indicate that five out of eight cases developed recurrences at local site or as pleural dissemination within 2 years of surgery [7]. The remarkable elevation of serum CEA value, which was observed in this case, did not seem to be common in papillary thymic carcinoma in the literature, while positive immunoreactivity of this antigen was sporadically reported [3]. More cases need to be accumulated to define the clinical features of this type of thymic carcinoma.

Not only for the rarity of the histology, the current case was characterized for the highly aggressive invasiveness to the sternum. The tumor destroyed the sternum and the costal cartilages with osteolytic change and reached subcutaneous fat tissue. Interestingly, any other adjacent organs such as great vessels, pericardium, and lung were not involved. As long as we searched similar cases in the literature, there was a case with thymic carcinoma minimally invading into the sternum [8]. However, it seemed to be rare that this disease represents the massive sternal invasion as seen in this case.

Resection of the sternum often requires skeletal and/or soft tissue reconstruction with artificial materials or a muscle flap. We discussed this issue with a plastic surgeon preoperatively and planed a pectoralis major muscle flap for an anterior chest wall reconstruction if it was necessary. Consequently, reconstruction was not indicated in this patient because sufficient stability and rigidity of the chest wall were maintained.

## Conclusion

We described the clinical and pathologic features of this extremely rare thymic papillary adenocarcinoma that invaded into subcutaneous tissue penetrating the sternum.

## Consent

Written informed consent was obtained from the patient for publication of this case report and any accompanying images. A copy of the written consent is available for review by the Editor-in-Chief of this journal.

## Abbreviations

CT: Computed tomography; FDG: 18 F-fluorodeoxy glucose; PET: Positron emission tomography; CEA: Carcino-embryonic antigen; AFR: Alpha-fetoprotein; beta-HCG: Beta-human chorionic gonadotropin; SCC: Squamous cell carcinoma antigen.

## Competing interests

The authors have declared that no conflict of interests exists.

## Authors’ contributions

IY analyzed and interpreted the patient data. JO performed the literature review, and was a major contributor in writing the manuscript. YT made a pathological diagnosis and prepared pathological figures. AF, TT, and TK helped surgery, encouraged to complete the work. MH supervised the data and the final editing of the manuscript. All authors read and approved the final manuscript.
